# Detecting *Plasmodium falciparum* in community surveys: a comparison of Paracheck Pf® Test and ICT Malaria Pf® Cassette Test to polymerase chain reaction in Mutasa District, Zimbabwe

**DOI:** 10.1186/s12936-020-03536-7

**Published:** 2021-01-06

**Authors:** Nobert Mudare, Zvifadzo Matsena-Zingoni, Aramu Makuwaza, Edmore Mamini, Shungu S. Munyati, Lovemore Gwanzura, Nicholas Midzi, Susan L. Mutambu, Peter Mason, Tamaki Kobayashi, Sungano Mharakurwa

**Affiliations:** 1 Africa University, Mutare, Zimbabwe; 2 National Institute of Health Research, Harare, Zimbabwe; 3 Biomedical Research and Training Institute, Harare, Zimbabwe; 4 University of Zimbabwe, College of Health Sciences, Institute of Continuing Health Education, Harare, Zimbabwe; 5 Johns Hopkins Bloomberg School of Public Health, Baltimore, MD USA

**Keywords:** *Plasmodium falciparum*, RDT, PCR, Microscopy, Zimbabwe, Honde valley

## Abstract

**Background:**

Microscopy and rapid diagnostic tests (RDTs) are the main techniques used to diagnose malaria. While microscopy is considered the gold standard, RDTs have established popularity as they allow for rapid diagnosis with minimal technical skills. This study aimed to compare the diagnostic performance of two *Plasmodium falciparum* histidine-rich protein 2 (PfHRP2)-based RDTs (Paracheck Pf® Test (Paracheck) and Malaria Pf™ ICT (ICT)) to polymerase chain reaction (PCR) in a community survey.

**Methods:**

A cross-sectional study was conducted between October 2012 and December 2014 in Mutasa District, Manicaland Province, eastern Zimbabwe. Households were randomly selected using satellite imagery, and 224 households were visited. Residents present in the household on the date of the visit were recruited for the study. Participants of all age groups from the selected households were screened with Paracheck and ICT RDTs in parallel. Dried blood spots (DBS) and thin and thick smears were collected. Parasite DNA extracted from the DBS was subjected to nested PCR targeting the *Plasmodium* cytochrome b mitochondrial gene. Data analysis was performed using the Cohen’s Kappa test to determine the interrater agreement and the sensitivity and specificity of the diagnostic test were reported.

**Results:**

Results from a total of 702 participants were analysed. Most were females, 397 (57%), and the median age of participants was 21 years with an interquartile range of 9–39 years. Of those who were screened, 8 (1.1%), 35 (5.0%), and 21 (2.9%) were malaria parasite positive by microscopy, RDT and PCR, respectively. Paracheck and ICT RDTs had a 100% agreement. Comparing RDT and PCR results, 34 participants (4.8%) had discordant results. Most of the discordant cases were RDT positive but PCR negative (n = 24). Half of those RDT positive, but PCR negative individuals reported anti-malarials to use in the past month, which is significantly higher than reported anti-malarial drug use in the population (*p* < 0.001). The participant was febrile on the day of the visit, but relying on PfHRP2-based RDT would miss this case. Among the diagnostic methods evaluated, with reference to PCR, the sensitivity was higher with the RDT (52.4%) while specificity was higher with the microscopy (99.9%). The positive predictive value (PPV) was higher with the microscopy (87.5%), while the negative predictive values were similar for both microscopy and RDTs (98%). Overall, a strong correlated agreement with PCR was observed for the microscopy (97.9%) and the RDTs (95.2%).

**Conclusions:**

Paracheck and ICT RDTs showed 100% agreement and can be used interchangeably. As malaria transmission declines and Zimbabwe aims to reach malaria elimination, management of infected individuals with low parasitaemia as well as non-*P. falciparum* infection can be critical.

## Background

Malaria presents a major health threat in sub-Saharan Africa, and Zimbabwe is not an exception. Transmission intensity of malaria in Zimbabwe is largely affected by the annual rainfall, and Manicaland Province, where the highest annual rainfall is reported, is the province most affected by malaria [1]. Through the support of various funding agencies, malaria control interventions in Zimbabwe were scaled-up [2]. Interventions include vector control, such as the distribution of insecticide-treated nets and indoor residual spraying (IRS), provision of artemisinin-based combination therapy, improved diagnostic capacity, and support for surveillance, monitoring and evaluation [1, 2]. Following the report of a high prevalence of insecticide resistance among local vectors [3], the Zimbabwe National Malaria Control Programme switched from pyrethroids to an organophosphate for the 2014 IRS campaign. The switch resulted in a 38% decrease in the incidence of malaria at health facilities in the sprayed wards, compared to the malaria incidence during the prior 24 months [4].

Despite the increased effort of national and international organizations, malaria remains a public health burden in high burden provinces in Zimbabwe, such as Manicaland [1]. In an area of declining malaria transmission, such as Manicaland Province, strategies to further decrease malaria transmission is critical. In the hope of a world free of malaria, the World Health Organization (WHO) urges malaria endemic countries to implement three pillars of strategies: Pillar 1. Ensure universal access to malaria prevention, diagnosis and treatment; Pillar 2. Accelerate efforts towards elimination and attainment of malaria-free status; and Pillar 3. Transform malaria surveillance into a core intervention [5]. When implementing the pillar 1 strategy, it is critical to optimise case detection and management at the community level [6]. To identify individuals who carry malaria parasites, rapid diagnostic tests (RDTs) are widely used because of its ease of use and promptness. *Plasmodium falciparum* histidine-rich protein 2 (PfHRP-2)-based RDTs are used for parasitological confirmation in sub-Saharan Africa, including Zimbabwe. The ICT Malaria-Pf® Cassette Test and Paracheck-Pf® Test are available in the Zimbabwe. At the time of the study, the Paracheck-Pf was registered in Zimbabwe, while ICT Malaria Pf Cassette was pending registration subject to field testing.

One of the WHO’s recommended strategies is to screen the community members even if an individual does not have malaria signs or symptoms. Malaria screening on the community can give us important epidemiological data to understand the prevalence of the infection in the community and how best to plan the future malaria control or elimination strategy. The present study compared the community-based diagnostic performance of two PfHRP-2 based RDTs and microscopy against PCR as a reference.

## Methods

### Study area

Mutasa District has a hot, temperate climate and high rainfall pattern ideal for vector breeding and malaria transmission. Perennial streams and gravity-fed irrigation channels span the low-lying *Honde Valley* part of the district, creating breeding sites for malaria vectors, mosquitoes. Malaria outbreaks have been a common occurrence in the district over the past few years despite the district meeting set targets for prevention interventions like indoor residual spraying (IRS) with pyrethroids and long-lasting insecticidal nets distribution. It is interesting, however, to note that National Malaria Control Program statistics reveal that Manicaland Province, mainly in Mutasa District, has become and remains a problem zone for the country, with a malaria incidence of 181/1000 in 2010, contributing a third to national malaria cases and deaths annually. This is despite the province meeting set targets of preventative and control interventions [7]. Mutasa District is located in the eastern part of Zimbabwe and shares its border with Mozambique. Malaria transmission is highly seasonal, peaking during the rainy season between November and April, and the main vector is *Anopheles funestus* [3]. Honde Valley, where the study was conducted, is home to large-scale tea estates, as well as small and subsistence farmers.

### Sampling method

The study was a part of a cross-sectional community survey conducted by the Southern and Central Africa International Centre of Excellence for Malaria Research and the study design and procedures were described previously [8]. Briefly, using satellite imagery, representative 1 km^2^ geographical grids were selected. Households within each grid were enumerated, and each household was visited once.

### Sample size

A total of 702 participants were enrolled in the study from 224 households, enabling detection of a minimum 5% difference in sensitivity between diagnostic tests with 95% confidence and 80% study power. All age groups were enrolled following requisite informed consent.

### Data collection

All consenting residents from selected households were enrolled in the study from October 2012 to July 2014. Signed informed consent was sought from all study participants, with parental/guardian permission for all children and child assent for children younger than 16 years of age. The socio-demographic characteristics and clinical data of the participants were collected using a structured questionnaire.

The temperature was taken with an ear thermometer (Braun, Thermoscan), and a tympanic temperature above 38 °C was considered to be a fever. Finger-prick blood samples were collected for thick and thin smears, preparation of dried blood spots (DBS) and two RDTs (Paracheck Pf® (Orchid Biomedical Systems, India) and ICT Malaria P.f. (ICT DIAGNOSTICS, South Africa). Individuals positive by RDTs were treated for malaria according to the Zimbabwean Ministry of Health guidelines [9]. The thin films were fixed in methanol after air-drying and stained with 3% Giemsa solution for 30 min. Thin and thick films were read by two experienced laboratory technicians, and the result was considered negative if no parasites were seen after examination of 1000 white blood cells at 100 × magnification.

The technicians were blinded to the results of the RDTs as well as the results by the other microscopist. In cases where the results were discordant, a third expert reader was used, and the results of the third expert reader were considered final. The DBS were transported to the National Institute of Health Research molecular laboratory in Harare for storage and PCR analysis.

### PCR detection of malaria infection

DNA was extracted from the DBS using the Chelex method as previously described [8]. Extracted DNA was subjected to a nested PCR assay targeting the mitochondrial cytochrome b gene (*cytb*) conserved in the major human *Plasmodium* species, using the primer sequences and cycling conditions previously reported [10]. The correct size PCR amplicon (815 bp) was confirmed using a 1% agarose gel, viewed under the UV transilluminator and analysed using FIRE-READER XS-D 55-20 M (UVITEC, Cambridge, UK).

### Data analysis

Descriptive statistics were reported as frequencies and percentages for categorical variables and median and interquartile range (IQR) for continuous variable. To compare differences in the prevalence of malaria parasitaemia between the diagnostic methods and covariates, the independent Chi-square was applied. The Cohen’s Kappa test was used to determine the interrater agreement between diagnostic tests. We estimated the area under the ROC curve of the RDTs and microscopy. The level of significance was set at 5%. The analysis was conducted using Stata 15.1.

## Results

A total of 702 participants from 224 households agreed to participate and were screened using two PfHRP2-based RDTs between October 2012 and July 2014 (Table 1). More than half of the participants were female (n = 397; 57%), majority were aged above 25 years (n = 292; 41.7%) and most of the participants were enrolled in 2013 (n = 353; 50.3%0). The overall median age was 21 years with an IQR of 9–39 years.

**Table 1:** Distribution of malaria prevalence in the study population based on age and gender stratified by the diagnostic tests in Mutasa District between 2012 and 2014 (N = 702)

Variable	Category	Diagnostic tests				Total (column %)
		Microscopy n(%)	Paracheck n(%)	ICT n(%)	PCR n(%)	
Malaria prevalence		8 (1.14)	35 (4.99)	35 (4.99)	21(2.99)	
Gender	Male	4 (0.57)	23 (3.28)	23 (3.28)	12 (1.71)	305 (43.45)
	Female	4 (0.57)	12 (1.71)	12 (1.71)	9 (1.28)	397 (56.55)
	Chi^2^ value	0.1414	7.4334	7.4334	1.6525	
	Chi^2^ p-value	0.707	**0.006**	**0.006**	0.199	
Age groups	< 5 years	1(12.5)	6(17.14)	6(17.14)	5(23.81)	115 (16.41)
	5–15 years	4(50.0)	10(28.57)	10(28.57)	5(23.81)	163 (23.25)
	16–25 years	1(12.5)	12(34.29)	12(34.29)	5(23.81)	131 (18.69)
	> 25 years	2(25.0)	7(20.0)	7(20.0)	6(28.57)	292 (41.65)
	Chi^2^ value	3.2697	9.4039	9.039	1.9199	
	Chi^2^ p-value	0.352	**0.024**	**0.024**	0.589	
Year enrolled	2012	2(25.0)	5(14.29)	5(14.29)	8(38.10)	117 (16.67)
	2013	3(37.5)	21(60.0)	21(60.0)	8(38.10)	353 (50.28)
	2014	3(37.5)	9(25.71)	9(25.71)	5(23.81)	232 (33.05)
	Chi^2^ value	0.6487	1.4163	1.4163	7.1629	
	Chi^2^ *p*-value	0.723	0.493	0.493	**0.028**	

^*^Bivariate tests were performed with reference to the malaria negative group; Bold-faced *p*values were significant at 5%

The prevalence of *Plasmodium* parasites detected by microscopy was 1.14% (n = 8) of which seven were *Plasmodium falciparum* mono-infections and one *Plasmodium ovale* mono-infection. The two PfHRP2-based RDTs, Paracheck-Pf® and ICT, detected the same individuals as PfHRP2-positive giving an equal malaria prevalence of 4.99% (n = 35). The amount of agreement of the results between the Paracheck-Pf® and ICT was a 100% amount which indicated that the results were not determined randomly (*p* < 0.001). The prevalence of *Plasmodium* infection based on PCR was 2.99% (n = 21). Multiple comparison of the malaria prevalence estimates based on RDT (4.99%), PCR (2.99%) and microscopy (1.14%) showed that there was no significant difference between RDT *vs* PCR estimates (*p* = 0.4705); marginal difference between RDT *vs* microscopy estimates (*p* = 0.0973) and no significant difference between PCR and microscopy estimates (*p* = 0.3124).

A contingency table displaying the numbers of individuals by RDT test result and PCR result showed that there were 34 individuals with discordant malaria results by the two tests. Of these 10 (29.4%) were PCR malaria positive and RDT malaria negative of which two were microscopy positive, one was *P. ovale* mono-infection and seven were *P. falciparum* mono-infection with very low parasitaemia (12 parasites/μl) by thick smear. The other 24 (70.6%) were PCR malaria negative and RDT malaria positive, and all were microscopy negative. Half of these individuals (n = 12) reported anti-malarial use within the past month, which was significantly higher than anti-malarial use among PCR and RDT negative participants (10% (68/654), *p* < 0.001).

**Fig. 1:**
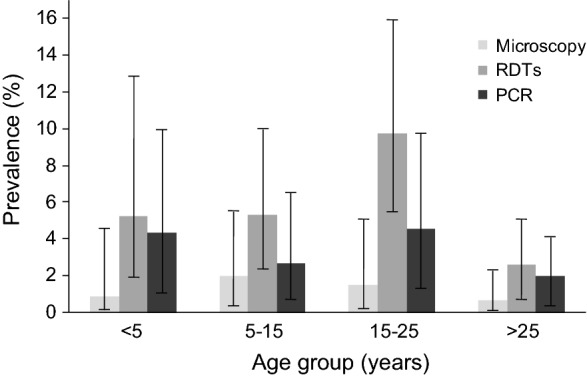
*P. falciparum* infection prevalence by age-group and malaria detection test

Parasite prevalence varied across age groups (Fig. 1). Those aged below 5 years had the highest malaria infections based on PCR results (23.81%); those aged 5–15 years had the highest malaria infections based on microscopy results (50%); and those aged 16–25 years had highest malaria infections based on RDT results (34.29%) and these results were statistically significant as the error bars were not overlapping. The bivariate tests further showed that there was no association between microscopy malaria infection status and gender (*p* = 0.707) or age groups (*p* = 0.353); there was a significant association between RDT malaria infection and gender (*p* = 0.006) or age groups (*p* = 0.025); and, there was a significant association between PCR malaria infection and year of enrolment (*p* = 0.028).

Malaria-positive participants bore infections with parasite densities ranging from 8–7256 and 16–7032 asexual parasites/µl of peripheral blood by first and second microscopist, respectively. In this community, the prevalence of fever was low. Of the 702 participants, 699 had tympanic temperature recorded on the day of the visit, and only 6 (0.9%) were febrile. None of the six febrile individuals was positive by RDTs, but one had *P. ovale* mono-infection by microscopy, and the presence of parasite DNA was confirmed by PCR.

The performance of the diagnostic tests with reference with each other are summarized in Table 2. Among the diagnostic methods evaluated, with reference to PCR, the sensitivity was higher with the RDT (52.4%) while specificity was higher with the microscopy (99.9%). The positive predictive value (PPV) was higher with the microscopy (87.5%) while the negative predictive values were similar for both microscopy and RDTs (98%). With reference to microscopy, the sensitivity (87.5%), specificity (98%) and PPV (33.3%) were higher with the PCR while NPV was higher with the RTD (99.6%). With reference to RDTs, the sensitivity was higher with the PCR (31.4%) while specificity was higher with the microscopy (99.6%). The PPV was higher with the microscopy (62.5%) while the NPV was higher for the PCR (96.5%). The highest ROC area was observed for PCR with reference to microscopy (92.7%). Overall, a perfectly correlated agreement with PCR was observed with the microscopy (97.9%) and the RDTs (95.2%). Moreover, a perfectly correlated agreement with RDTs was observed with the microscopy (95.3%).

**Table 2:** Performance of the diagnostic tests with reference to each other

	PCR as reference		Microscopy as reference		RDT as reference	
Parameters	Microscopy % (95%CI)	RDTs % (95%CI)	PCR % (95%CI)	RDT % (95%CI)	PCR % (95%CI)	Microscopy % (95%CI)
Sensitivity	33.3 (14.6–57)	52.4 (29.8–74.3)	87.5 (47.3–99.7)	62.5 (24.5–91.5)	31.4 (16.9–49.3)	14.3 (4.81–30.3)
Specificity	99.9 (99.2–100)	96.5 (94.8–97.7)	98(96.6–98.9)	95.7(93.9–97.1)	98.5(97.3–99.3)	99.6 (98.7–99.9)
PPV	87.5( 47.3–99.7)	31.4(16.9–49.3)	33.33 (14.6–57)	14.3 (4.81–30.3)	52.4(29.8–74.3)	62.5(24.5–91.5)
NPV	98 (96.6–98.9)	98.5(97.3–99.3)	99.2(99.2–100)	99.6(98.7–99.9)	96.5(94.8–97.7)	95.7(93.9–97.1)
ROC area	0.666 (0.563–0.769)	0.744(0.635–0.854)	0.927(0.805–1)	0.791 (0.611–0.97)	0.65 (0.571–0.728)	0.569 (0.51–0.628)
Percentage agreement of the tests	97.86%	95.16%	97.86%	95.30%	95.16%	95.30%
Cohan Kappa value	0.4741	0.3693	0.4741	0.2181	0.3693	0.2181
Kappa p-value	< 0.001	< 0.001	< 0.001	< 0.001	< 0.001	< 0.001

*PPV * positive predictive value, *NPV* negative predictive value, *PCR* polymerase chain reaction, *RDT* rapid diagnostic test

## Discussion

The prevalence of afebrile *P. falcipar*um parasite carriers in Mutasa District in Zimbabwe was low in an area of seasonal *P. falciparum* transmission. The current standard malaria diagnostics is based on microscopy and rapid diagnostic tools (RDT) that have the lower detection limit of approximately 50–100 parasites per µL of blood, much higher than the WHO-recommended limit of ≤ 200 IE/100 µL [11, 12]. Using the two different PfHRP2-based RDTs, the prevalence was 5%, which was significantly higher than the prevalence by microscopy. By comparing RDT results with PCR results, there was a significant discordance: approximately two-thirds of RDT-positive individuals had no detectable parasite DNA by PCR, and about half of PCR-positive individuals were missed by RDTs. Albeit the current study scope did not include assessing the presence of HRP2 deleted parasitaemia, further studies are recommended to ascertain the prevalence of such parasite variants as they can also contribute RDT false negatives. The age category with the highest parasite prevalence by RDT or PCR was adolescents between 15–25 years of age.

In recent years, the use of RDT expanded from medical facilities to community testing. Integrated community case management, a strategy to extend care for common causes of childhood mortality and morbidity (pneumonia, diarrhoea, and malaria), is provided by the community by health workers [13], and in some cases implementation of mass screening and testing [14]. In this study, two-thirds (N = 24) of RDT positive individuals did not have detectable *Plasmodium* DNA in the capillary blood. It has been reported that the RDTs can remain positive for more than five weeks after successful treatment because of the prolonged persistence of PfHRP2 antigen in the circulation after parasite clearance [15]. Half of the RDT-positive and PCR-negative participants in the present study reported recent use of anti-malarials, and such information would be informative if RDTs are to be used for test-and-treat strategies. The two RDTs tested in this study were PfHRP2-based RDTs, but the only febrile *Plasmodium* infection identified was due to *P. ovale*, which was missed by both RDTs tested. Transmission of *P. ovale* in this area has been previously reported, albeit at very low incidence [16]*.* Supplementing pLDH-based RDT at clinical settings may improve the detection of non-falciparum infections.

PfHRP2-based RDT sensitivity could be affected by the genetic diversity of the target antigen in some settings [17], and deletion of the *Pfhrp2/3* gene has been reported in sub-Saharan African countries [18]. There was was no information about *pfhrp2/3* deletions in the study area; therefore, the possibility of false-negative RDTs due to target gene deletions could not be ruled out. For the RDT negative but PCR positive samples, microscopy data were examined and nine out of ten were confirmed as microscopy negative and the positive sample had low parasitaemia, below the typical detection limit of RDTs (200 parasite/μL) [19].

Microscopists classified a slide as negative after 1,000 white blood cells, providing a theoretical detection limit of 8 parasites/μL. A well-trained microscopist with long years of experience has superior detection limit compared to currently available RDT [20]; therefore, it was considered that false RDT-negative results may be more likely due to a low parasitaemia than *pfhrp2/3* deletions. The specificity and sensitivity of the two RDTs (Paracheck P.f®. and ICT RDT kits) and microscopy were estimated relative to Cytochrome b PCR. PCR is more sensitive and specific than an examination of thick or thin blood smears, particularly in cases with low parasite rates or mixed infections [21–27]. PCR is also more sensitive than the various dipstick assays [27]. Therefore, in this study, the PCR result was used as a reference for the malaria parasite present in the blood sample. The samples in this study were from mainly asymptomatic participants, who are known to carry lower parasitaemia than symptomatic cases [28], and it is possible that most infected individuals had low levels of parasitaemia below the detection limit of RDTs. Although the majority of studies have shown that PCR is both sensitive and specific for the detection of malaria, there are limitations that can affect the accuracy of the method. Selection of appropriate primers, methods used for collection and storage of blood samples, and extraction methods can all affect PCR performance. Jelinek et al*.* [29] reported that the sensitivity of PCR was as much linked to parasite density as was microscopy. They found that sensitivity of PCR was affected by both parasite density and by geographic differences in parasite populations. Other studies have reported that PCR may occasionally yield false negative results [30–32]. Barker et al*.* [33] carefully analysed discrepancies between microscopy and PCR, and although true false negative PCR results did occur, the majority of discrepancies resulted from problems with microscopy. Most recently, Scopel et al*.* [34] determined that use of DNA extracted from thick blood smears resulted in poor detection of malaria parasites, particularly with parasite densities less than 20/μL.

## Limitations

The current study did not collect information about *pfhrp2/3* deletions in the study area. Therefore, the possibility of false-negative RDTs due to target gene deletions could not be ruled out. Further studies are recommended to examine pfhrp2/3 deletions in the area.

## Conclusions

In screening community-based individuals for *P. falciparum* infection, two RDT brands, Paracheck- Pf and ICT, detected the same number of infections with 100% agreement and could be used interchangeably. However, a high proportion of false-positive results was observed, presumably due to persisting antigenaemia. Based on microscopy results, RDT-negative, PCR-positive individuals had low-level parasitaemia, but further studies are needed to rule out the possibility of *pfhrp2/3* deletions. This study also highlights the fact that there is a risk of missing non-falciparum infections when relying on PfHRP2-based RDTs.

## Abbreviations

IRS: Indoor residual spraying
RDT: Rapid diagnostic test
PfHRP2L: *Plasmodium falciparum* Histidine rich protein 2
PCR: Polymerase chain reaction
